# Assessment of the quality of end-of-life care: translation and validation of the German version of the “Care of the Dying Evaluation” (CODE-GER) - a questionnaire for bereaved relatives

**DOI:** 10.1186/s12955-020-01473-2

**Published:** 2020-09-22

**Authors:** Annika Vogt, Stephanie Stiel, Maria Heckel, Swantje Goebel, Sandra Stephanie Mai, Andreas Seifert, Christina Gerlach, Christoph Ostgathe, Martin Weber

**Affiliations:** 1 Interdisciplinary Palliative Care Unit, III. Department of Medicine, University Medical Center of the Johannes Gutenberg University of Mainz, Langenbeckstr.1, 55131 Mainz, Germany; 2 Hannover Medical School, Institute for General Practice, Carl-Neuberg-Straße 1, 30625 Hannover, Germany; 3 Friedrich-Alexander-Universität Erlangen- Nürnberg (FAU), Department of Palliative Medicine, CCC Erlangen –EMN, Krankenhausstraße 12, 91054 Erlangen, Germany; 4 Centre for Educational Research and Teacher Training (PLAZ), Paderborn University, Warburger Straße 100, 33098 Paderborn, Germany

**Keywords:** Terminal care, Quality of health care, Proxy, Hospital, Validation studies, outcome assessment

## Abstract

**Background:**

International studies indicate deficits in end-of-life care that can lead to distress for patients and their next-of-kin.

The aim of the study was to translate and validate the “Care of the Dying Evaluation” (CODE) into German (CODE-GER).

**Methods:**

Translation according to EORTC (European Organisation for Research and Treatment of Cancer) guidelines was followed by data collection to evaluate psychometric properties of CODE-GER. Participants were next-of-kin of patients who had died an expected death in two hospitals. They were invited to participate at least eight, but not later than 16 weeks after the patient’s death. To calculate construct validity, the Palliative care Outcome Scale (POS) was assessed. Difficulty and perceived strain of answering the questionnaire were assessed by a numeric scale (0–10).

**Results:**

Out of 1137 next-of-kin eligible, 317 completed the questionnaire (response rate: 27.9%). Data from 237 main sample participants, 38 interraters and 55 next-of-kin who participated for repeated measurement were analysed. Overall internal consistency, α = 0.86, interrater reliability, ICC (1) = 0.79, and retest-reliability, ICC (1, 2) = 0.85, were good. Convergent validity between POS and CODE-GER, r = −.46, was satisfactory. A principal component analysis with varimax rotation showed a 7-factor solution. Difficulty, M = 2.2; SD ± 2.4, and perceived strain, M = 4.1; SD ± 3.0, of completing the questionnaire were rather low.

**Conclusion:**

The results from the present study confirm CODE-GER as a reliable and valid instrument to assess the quality of care of the dying person. More over our study adds value to the original questionnaire by proposing a deepened analysis of obtained data. The development of seven subscales increases its potential for further surveys and research.

**Trial registration:**

This study was registered retrospectively on the 25th of January 2018 at the German Clinical Trials Register (DRKS00013916).

## Background

According to the founder of modern palliative care (PC) Cicely Saunders, physical, psychological, social and spiritual needs have to be considered when caring for dying patients and their families [1]. Despite the clear need for PC, not all dying patients can be treated on specialized wards due to limited access or space [2, 3]. Therefore, it is of great importance to extend the principles of PC to any wards where people die.

It is equally important to assess the current state of quality of care (QOC) on these wards, and to identify unmet needs of patients and their next-of-kin. While the patients themselves are often unable to provide information about the perceived quality of their care, their next-of-kin can evaluate the last days of their loved ones [4]. They are not only providers of support to the patients, but also recipients of PC themselves [5]. Therefore an instrument which assesses the care given to the patient, but also to their next-of-kin, is crucial to represent holistic care at the end of life. To the best of our knowledge the only instrument that assesses a similar construct in German is the “Quality of Death and Dying” (QoDD) which was validated by some of this study’s authors [6]. However, QoDD surveys the quality of death and not the quality of care given to the dying patient.

A suitable instrument for this purpose, the “Care of the Dying Evaluation” (CODE™) was developed by selecting key indicators from the rather long “Evaluation Care and Health Outcomes – for the Dying” (ECHO-D) [7]. CODE™ is a self-assessment questionnaire which retrospectively evaluates the QOC in the last 2 days of a patient’s life by surveying next-of-kin. Twenty-eight core items cover different aspects of QOC (care received from healthcare team, symptom control, communication with the healthcare team, emotional and spiritual support, circumstances surrounding death). Verbal anchors represent a 5-point (0–4), 4-point (0–3) or 3-point (0–2) Likert scale. The higher the value, the better the QOC [7]. Three key composite scales which are represented by 12 of the 28 core items, survey “Environment”, “Care” and “Communication”. The items were initially assigned to the scales based on theoretical assumptions. Furthermore, CODE™ captures overall impression concerning treatment with respect and dignity by doctors and nurses as well as support of relatives. Ten items assess demographic or disease-related information [7].

CODE™ has so far been validated for the United Kingdom [7]. Internal consistencies of the key composite scales were good (α = 0.79–0.89). Test-retest-reliability was moderate to good [7]. A recent systematic review on tools measuring quality of death, dying and care completed after death identified CODE™ as an instrument with promising strong psychometric properties, which would benefit from further development and validation [8].

## Methods

The aim of this study was to provide a German version of CODE™ (CODE-GER) and to evaluate its psychometric properties.

### Translation process, pretesting and questionnaire adaption

Between 01/2013 and 04/2013, CODE™ was translated forward and backward according to EORTC guidelines [9]. To assess content validity, ‘think aloud’ interviews and verbal probing took place with 15 next-of-kin of deceased patients at 2 PC units (Mainz: *n* = 7; Erlangen: *n* = 8). Results from this pilot testing were discussed by an expert panel with expertise in PC. No items were evaluated as inappropriate, confusing or embarrassing. The questionnaire itself was rated as useful. Adaptions to the wording and formal structure were made. The 28 core items on QOC were maintained without modification. One item (recommendability of ward) was added to the overall section (originally three items). The 28 core items on QOC as well as the overall impression questions are shown in Table 1. Three items (type of ward, nationality of caregiver, amount of days on the ward where the patient had died) were added to the demographic section (originally 10 items compared to the original English version). The items of the resulting questionnaire CODE-GER used in this study are shown in Table 1.

**Table 1:** CODE GER - items included in psychometric analyses and overall impression questions

Item CODE™	Short Description	Verbal Anchors and Rating Scale
There was enough help available to meet his/her personal care needs, such as washing, personal hygiene and toileting needs.	Nursing care- personal care needs	0 = strongly disagree 1 = disagree 2 = neither agree nor disagree 3 = agree 4 = strongly agree
There was enough help with nursing care, such as giving medicines and helping him/her find a comfortable position in bed.	Nursing care – medicines and comfortable position	0 = strongly disagree 1 = disagree 2 = neither agree nor disagree 3 = agree 4 = strongly agree
The bed area and surrounding environment was comfortable for him/her.	Whether the bed area was comfortable	0 = strongly disagree 1 = disagree 2 = neither agree nor disagree 3 = agree 4 = strongly agree
The bed area and surrounding environment had adequate privacy for him/her.	Whether the bed area had adequate privacy	0 = strongly disagree 1 = disagree 2 = neither agree nor disagree 3 = agree 4 = strongly agree
In your opinion, how clean was the ward area that s/he was in? ^a^	Whether ward was clean	0 = Not at all clean 2 = Fairly clean 4 = Very clean
Did you have confidence and trust in the nurses who were caring for him/her?	Confidence and trust in nurses	0 = No, not in any of the nurses 2 = Yes, in some of them 4 = Yes, in all of them
Did you have confidence and trust in the doctors who were caring for him/her?	Confidence and trust in doctors	0 = No, not in any of the doctors 2 = Yes, in some of them 4 = Yes, in all of them
The nurses had time to listen and discuss his/her condition with me.	Time of nurses to listen and discuss the patients’ condition	0 = strongly disagree 1 = disagree 2 = neither agree nor disagree 3 = agree 4 = strongly agree
The doctors had time to listen and discuss his/her condition with me.	Time of doctors to listen and discuss the patient’s condition	0 = strongly disagree 1 = disagree 2 = neither agree nor disagree 3 = agree 4 = strongly agree
In your opinion, during the last 2 days, did s/he appear to be in pain?	Whether patient had pain	0 = Yes, all of the time 2 = Yes, some of the time 4 = No
In your view, did the doctors and nurses do enough to help relieve the pain?	Whether HCT did all they could to relieve pain	0 = No, not at all 2 = Yes, some of the time 4 = Yes, all of the time 4 = Not applicable, s/he was not in pain
In your opinion, during the last 2 days, did s/he appear to be restless?	Whether patient was restless	0 = Yes, all of the time 2 = Yes, some of the time 4 = No
In your opinion, did the doctors and nurses do enough to help relieve the restlessness?	Whether HCT did all they could to relieve restlessness	0 = No, not at all 2 = Yes, some of the time 4 = Yes, all of the time 4 = Not applicable, s/he was not restless
In your opinion, during the last 2 days, did s/he appear to have a ‘noisy rattle’ to his/her breathing?	Whether patient had retained respiratory tract secretions	0 = Yes, all of the time 2 = Yes, some of the time 4 = No
In your view, did the doctors and nurses do enough to help relieve the ‘noisy rattle’ to his/her breathing?	Whether HCT did all they could to control respiratory tract secretions	0 = No, not at all 2 = Yes, some of the time 4 = Yes, all of the time 4 = Not applicable, s/he had no noisy rattle
During the last 2 days, how involved were you with the decisions about his/her care and treatment?	Involvement in decision-making	0 = Not involved 2 = Fairly involved 4 = Very involved
Did any of the healthcare team discuss with you whether giving fluids through a ‘drip’ would be appropriate in the last 2 days of life?	Whether discussion of giving fluids through a ‘drip’ took place	0 = No 4 = Yes
Would a discussion about the appropriateness of giving fluids through a ‘drip’ in the last 2 days of life have been helpful?	Whether discussion giving fluids through a ‘drip’ would have been helpful	0 = Yes 4 = No 4 = Not applicable, we had these type of discussions
Did the healthcare team explain his/her condition and/or treatment in a way you found easy or difficult to understand?	Difficulty of explanations of the patient’s condition	0 = They did not explain his/her condition or treatment to me 1 = Very difficult 2 = Fairly difficult 3 = Fairly easy 4 = Very easy
How would you assess the overall level of emotional support given to you by the healthcare team?	Emotional support to next-of-kin	0 = Poor 1 = Fair 3 = Good 4 = Excellent
Overall, his/her religious or spiritual needs were met by the healthcare team.	Whether HCT met overall religious spiritual needs of patient	0 = strongly disagree 1 = disagree 2 = neither agree nor disagree 3 = agree 4 = strongly agree
Overall, my religious or spiritual needs were met by the healthcare team.	Whether HCT met overall religious spiritual needs of next-of-kin	0 = strongly disagree 1 = disagree 2 = neither agree nor disagree 3 = agree 4 = strongly agree
Before s/he died, were you told s/he was likely to die soon?	Information about the soon death of the patient	0 = No 4 = Yes
Did a member of the healthcare team talk to you about what to expect when s/he was dying (e.g. symptoms that may arise)?	Information about what to expect during the dying process of the patient	0 = No 4 = Yes
Would a discussion about what to expect when s/he was dying have been helpful?	Whether a discussion about what to expect during the dying process would have been helpful	0 = No 4 = Yes 4 = Not applicable, we had these types of discussions
In your opinion did s/he die in the right place? ^a^	Whether patient died in the right place	0 = No, it was not the right place 2 = Not sure 4 = Yes, it was the right place
I was given enough help and support by the healthcare team at the actual time of his/her death	Support at actual time of death	0 = strongly disagree 1 = disagree 2 = neither agree nor disagree 3 = agree 4 = strongly agree
After s/he had died, did individuals from the healthcare team deal with you in a sensitive manner?	Sensitivity of HCT after death	0 = No 4 = Yes
**Overall Impression**		
How much of the time was s/he treated with respect and dignity in the last 2 days of life by doctors?	Whether patient was treated with respect and dignity by doctors	0 = Never 1 = Some of the time 3 = Most of the time 4 = Always
How much of the time was s/he treated with respect and dignity in the last 2 days of life by nurses?	Whether patient was treated with respect and dignity by nurses	0 = Never 1 = Some of the time 3 = Most of the time 4 = Always
Overall, in your opinion, were you adequately supported during his/her last 2 days of life?	Whether next-of-kin was adequately supported	0 = No 4 = Yes
How likely are you to recommend our ward to friends and family?	Whether next-of-kind would recommend ward to family/friends	0 = Extremely unlikely 1 = Unlikely 2 = Neither likely nor unlikely 3 = Likely 4 = Extremely likely

^a^= Item was deleted after psychometric analyses for the final version of CODE-GER

After completing the CODE-GER, participants additionally were asked to give the time they needed to fill in the questionnaires and one question on the difficulty and the perceived strain of completing the questionnaire. They used a scale from 0 (very easy/no strain) to 10 (very hard/high strain). Although these questions have not yet been validated formally they have been used in previous studies [10–12].

### Study population and data collection

The study was conducted at the two German university hospitals of Mainz (MZ) and Erlangen (E) on the following types of ward: intensive care, palliative care, internal medicine and neurology. A minimum number of 200 next-of-kin were planned to be included, as recommended for psychometric testing by Lienert and Raatz [13].

All consecutive patients who had died on these wards between 04/2016 and 03/2017 were included according to the following eligibility criteria:

- ≥ 18 years old
- stay ≥3 days on the ward where death occurred,
- expected death, based on physician’s judgment that the patient was soon to die, and the cause of death was not sudden.

To identify eligible patients, databases of all deaths on the predefined wards were electronically screened for criteria (a) and (b). Next, the responsible physicians were contacted personally to check for criterion (c). Next-of-kin data of patients were extracted from the electronic hospital information system. If more than one next-of-kin was registered, all of them were contacted to assure an inter-rater-population. Eligible next-of-kin were informed about the study and invited to take part by post at least eight, but not later than 16 weeks after the death. Next-of-kin were defined as family, friends or legal guardian. Through a postcard, which was sent to next-of kin, next-of-kin were able to inform the study team whether they wished to receive study information. If the corresponding box was ticked, a trained researcher phoned the next-of-kin to provide them with further information and to check for the following exclusion criteria: Under 18 years old; insufficient German language skills; no contact with patient in the last 2 days of life.

Eligible next-of-kin were asked whether they felt emotionally stable enough to participate in the study. After consent was given verbally over the phone, the study documents (detailed study information, informed consent form, CODE-GER, Palliative care Outcome Scale (POS) and a prepaid envelope) were sent to participants (T1). Participants were asked to tick a box on the informed consent form to indicate if they would be willing to repeat the survey (T2). Those who agreed received a second study pack 8 weeks after the first documents were completed. To determine interrater reliability, this first next-of-kin group was asked to provide contact details for additional relatives present during the last 2 days of the patient’s life. Additional next-of-kin underwent the same recruitment process as the first next-of-kin group, although the latter were called directly if phone numbers were provided.

### Description of questionnaires

All participants were asked to complete CODE-GER. In addition, participants completed the Palliative Care Outcome Scale (POS) for families. As there is no German instrument available regarding equivalent content, the content wise comparable tool POS (available as a validated German version) was chosen to allow for an approximate external criterion, since it assesses the convergent validity of CODE to some extent. POS is a 12-item self-assessment instrument that surveys for symptoms, concerns and psychosocial needs of patient and family in the past 3 days of the patient’s life. Answers are scored on a 0–4 Likert scale. Scores of items 1–10 can be summarized into a Total-Score (0–40). Higher scores are associated with higher distress [10].

### CODE-GER items and Total-score

Verbal anchors provided in the answering possibilities represent a 5-point (0–4), 4-point (0–3) or 3-point (0–2) Likert scale. In order to establish a unified rating scale from “0” to “4”, the following rules were defined: the highest possible answer, indicating high quality, was coded with “4”, while “0” was assigned to the lowest. Middle categories were represented by “2” (for more details see Table 1).

For further analysis values of single items were summed up according to their respective subscales. Next, these values were added up to form a Total-Score (0–104). A high Total-Score corresponds to high quality end-of-life care.

Items with more than 50% of missing values across all questionnaires were excluded from further analysis [14]. To minimize the effect of imputation, a maximum of 15% of missing items was tolerated and imputed by Expectation Maximization for interval variables per questionnaire [15]. Questionnaires with more than 15% missings were excluded from further analysis. Missing values for dichotomous variables were imputed by the mode of the corresponding item to ensure conformity with the rating scale.

### Data analysis

#### Psychometric properties

Since only 12 of the 28 core items of the original CODE TM questionnaire have been examined by factor analytic methods so far, we analyzed the 28 core items on QOC with an explorative factor analysis. In order to explain as much variance as possible in the data we conducted a principal component analysis (PCA) with varimax rotation. To test whether our data were suitable for PCA the Bartlett’s test and Kaiser-Meyer-Olkin Measure of Sampling Adequacy were carried out. The number of factors was determined by the Kaiser-Guttmann criterion (eigenvalues > 1), analysis of the scree plot and conceptual fit [16, 17].

#### Inclusion of items

Decisions on the assignment of items to a factor were based on the following criteria:

- higher Cronbach’s alpha if item was included (concerning the subscale)
- item to total correlation ≥0.4 [18]
- factor loading ≥0.3 [19]
- items that only load on one factor
- consistency between the item and the content of the factor

All criteria should be met. In doubtful cases criterion e) was pivotal.

#### Reliability

Internal consistency was measured with Cronbach’s alpha [20]. Values ≥0.7 are regarded as satisfactory [21].

Interrater reliability was calculated with intraclass correlation (ICC) estimates and their 95% confidence intervals (CI) for Total-Scores, based on a one-way random model (ICC (1)). For test-retest reliability, ICC was calculated with Total-Scores of T1 and T2 with a single rating, absolute agreement, two-way mixed-effects model (ICC (1, 2)) [22, 23]. An ICC < 0.5 indicated poor reliability, 0.5–0.75 moderate, 0.75–0.9 good and > 0.9 excellent [24].

#### Validity

Construct validity was assessed with convergent validity by a Pearson’s correlation coefficient between the Total-Scores of CODE-GER and POS. As low values in POS are associated with low distress, a negative correlation was expected.

#### Items of overall impression

To examine if items of overall impression (Table 1) represented the Total-Score, Pearson’s (r) or Spearman’s rank (rs) correlations were calculated according to the rating scale of the item. Correlations ≤0.3, > 0.3, > 0.7 are regarded as low, moderate and high, respectively [25].

#### Recruitment and demographic and disease-related information

Data on the recruitment of the study population and their demographic and disease-related information as well as the time needed to answer the CODE-GER were analyzed using descriptive statistics and frequency analysis.

All statistical analyses were performed using IBM SPSS Statistics 23 for Windows [26].

## Results

### Study population and data collection

**Fig. 1:**
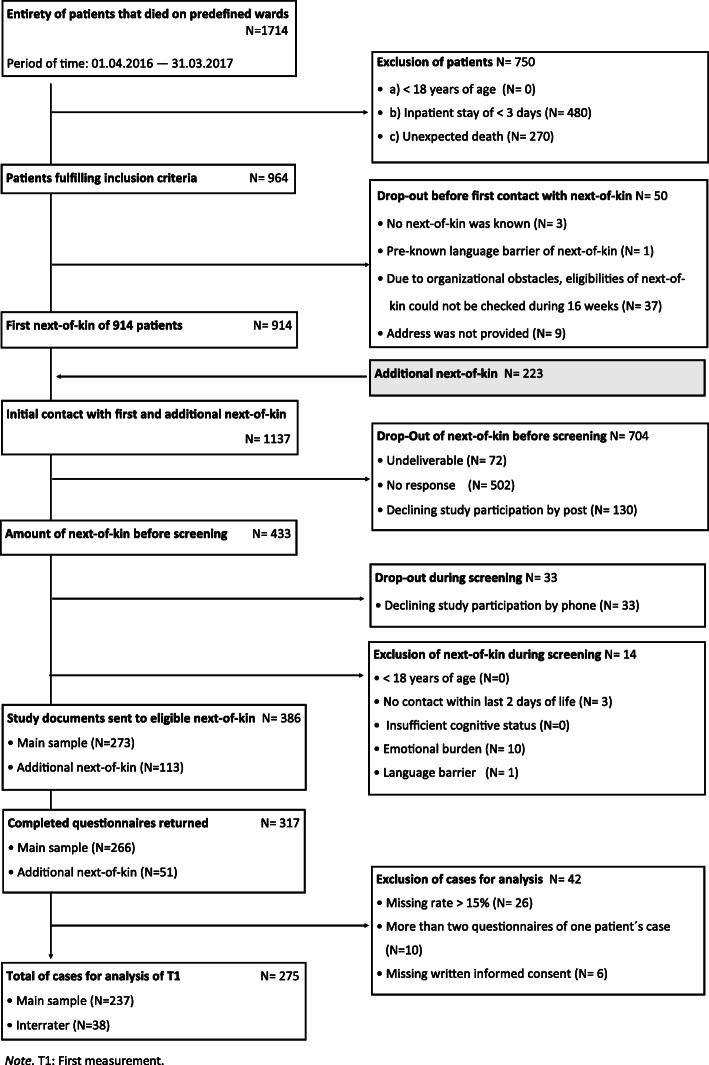
Flow-chart for study partcipation

A total of 1714 patients died during the recruitment period. According to criteria a–c 750 patients were excluded. Fifty patients and their next-of-kin dropped out before first contact. Eventually, 1137 next-of-kin were invited to participate in the study, comprising 914 next-of-kin initially contacted, and 223 additional next-of-kin. Before phone screening, 704 dropped out. During the screening, 33 next-of-kin declined participation, and 14 were excluded. Eventually, 317 of 386 eligible and approachable next-of-kin returned the study documents (overall response rate: 27.9%). For statistical analysis 42 cases were excluded. As a consequence of deleting cases of first measurement, seven cases of repeated measurement were excluded, leaving 55 out of 62 completed questionnaires for repeated measurement analysis. The main sample consisted of 237, the interrater group of 38 participants. Details of data collection including reasons for drop out and exclusion are shown in the flow chart of study participation (Fig. 1).

### Characteristics

Most participants were between 50 and 59 (29%) and 60 and 69 (24%) years, 65% were female. Most participants were either “husband/wife/partner” (43%) or “son/daughter” (41%). In total, 213 (90%) were German with no migrant background. As for religion, 81% were Christians, 0.5% were New Apostolic, and 18% had no religious affiliation. The most common diagnosis of the deceased patients was cancer (57%). The average length of inpatient stay was 13.7 days (SD ± 21.1; range = 3–276). Further subject characteristics are presented in Table 2.

**Table 2:** Characteristics of next-of-kin and information about patients

	Main sample (*N* = 237)	Interrater (*N* = 38)	T2 (*N* = 55)
	Number (%)	Number (%)	Number (%)
*Gender*			
Female	153 (65)	27 (71)	36 (66)
Male	84 (35)	11 (29)	19 (34)
*Age*			
20–29	6 (3)	4 (11)	2 (4)
30–39	20 (8)	–	5 (9)
40–49	34 (14)	10 (26)	10 (18)
50–59	69 (29)	15 (40)	17 (31)
60–69	56 (24)	5 (13)	15 (27)
70–79	33 (14)	2 (5)	5 (9)
80+	19 (8)	2 (5)	1 (2)
*Relation to patient*			
Husband / Wife / Partner	101 (43)	11 (30)	21 (38)
Son / Daughter	96 (41)	15 (39)	23 (42)
Brother / Sister	13 (6)	4 (10)	3 (5)
Son-in-law / Daughter-in-law	4 (2)	6 (16)	2 (4)
Parent	9 (3)	–	1 (2)
Friend	3 (1)	–	–
Other	11 (4)	2 (5)	5 (9)
*Nationality*			
Germany	213 (90)	35 (92)	52 (94)
Austria	1 (0.3)	–	–
Croatia	1 (0.3)	–	–
Greece	1 (0.3)	–	–
Italy	2 (1)	–	–
Missing	19 (8)	3 (8)	3 (6)
*Religion*			
Roman Catholic	101 (43)	18 (47)	22 (40)
Protestant	89 (38)	12 (32)	24 (44)
Muslim	–	–	–
None	43 (18)	6 (16)	8 (14)
New apostolic	2 (0.5)	1 (2.5)	1 (2)
Buddhist	–	1 (2.5)	–
Missing	2 (0.5)	–	–
*Main diagnosis of patient assessed by relative*^a^			
Cancer	134 (57)	24 (63)	35 (64)
Kidney disease	48 (20)	8 (21)	6 (11)
Heart failure	41 (17)	6 (16)	4 (7)
Stroke	33 (14)	5 (13)	7 (13)
COPD	19 (8)	3 (8)	4 (7)
Dementia	19 (8)	6 (16)	7 (13)
Motor neurone disease	2 (1)	–	–
Don’t know	5 (2)	–	1 (2)
Something else	51 (22)	12 (32)	13 (24)
*Ward on which patient had died*			
Palliative Care Unit	120 (51)	24 (64)	32 (58)
Internal medicine and neurology	60 (25)	7 (18)	10 (18)
Intensive care unit	57 (24)	7 (18)	13 (24)

^a^Multiple selection was possible. Percentage rates reflect the amount of one diagnosis per sample.; T2 = Participants of repeated measurement

### Missing values

Missing rates for items “whether health care team met overall religious spiritual needs of patient” and “whether health care team met overall religious spiritual needs of next-of-kin” were about 10% each. Missing rates for the remaining items ranged between 0.4 and 7.6%.

### Psychometric properties

Bartlett’s test (χ^2^(378) = 2839.3; *p* < .001) and Kaiser-Meyer-Olkin Measure of Sampling Adequacy (0.8) indicated suitability for PCA. Therefore, a PCA with varimax rotation was conducted. As the scree plot did not show a definite “knee” point, a 7-factor solution (Table 3) based on eigenvalues and its best conceptual fit was chosen. This solution explained 61.8% of the variance and included all core items on QOC. Items with critical values (shown in bold in Table 3) were analysed according to inclusion of items criteria (see Methods section).

**Table 3:** Item and Scale characteristics after PCA with varimax rotation

					Factor Loadings						
Subcales and short description of items	M	SD	Cronbachs’Alpha if item deleted per scale	Item Scale Correlation	F 1	F2	F 3	F 4	F 5	F 6	F 7
**1 Support and time of doctors and nurses** α = 0.85; 14.6% Variance explained											
Nursing care – medicines and comfortable position	3.8	0.6	0.82	0.68	0.73						
Nursing care- personal care needs	3.7	0.7	0.82	0.68	0.72						
Confidence and trust in doctors	3.6	0.9	0.83	0.57	0.67						
Time of doctors to listen and discuss the patient’s condition	3.5	0.9	0.82	0.69	0.66						
Whether HCT did all they could to relieve pain	3.9	0.5	0.84	0.54	0.62						
Whether HCT did all they could to control respiratory tract secretions	3.6	0.9	0.85	0.41	0.59						
Confidence and trust in nurses	3.7	0.8	0.84	0.51	0.56						
Whether HCT did all they could to relieve restlessness	3.6	0.9	0.83	0.56	0.47						
Time of nurses to listen and discuss the patients’ condition	3.5	0.8	0.83	0.57	**0.40**	**0.48**					
**2 Spiritual and emotional support** α = 0.82; 10.6% Variance explained											
Whether HCT met overall religious spiritual needs for patient	3.3	1.0	0.69	0.82		0.84					
Whether HCT met overall religious spiritual needs for next-of-kin	3.3	1.1	0.72	0.77		0.81					
Emotional support to next-of-kin	3.4	0.9	0.80	0.60		0.58					
Whether ward was clean^a^	3.7	0.8	**0.86**	0.43		0.50					
**3 Information and decision-making** α = 0.61; 8.5% Variance explained											
Difficulty of explanations of the patient’s condition	3.1	1.0	0.50	0.54			0.70				
Involvement in decision-making	3.0	1.4	0.43	0.55			0.66				
Whether discussion giving fluids through a ‘drip’ would have been helpful	3.2	1.6	**0.62**	**0.28**			0.62				
Whether discussion of giving fluids through a ‘drip’ took place	1.8	2.0	**0.62**	**0.34**			**0.52**				**0.41**
**4 Environment** α = 0.67; 8.2% Variance explained											
Whether the bed area had adequate privacy	3.6	0.8	0.37	0.65				0.85			
Whether the bed area was comfortable	3.5	0.9	0.54	0.51				0.78			
Whether patient died in the right place^a^	3.5	1.1	**0.81**	**0.34**				0.50			
**5 Information about dying process** α = 0.68; 7.6% Variance explained											
Whether a discussion about what to expect during the dying process would have been helpful	2.9	1.8	0.51	0.55					0.75		
Information about what to expect during the dying process of the patient	2.2	2.0	0.56	0.52					0.69		
Information about the soon death of the patient	3.4	1.4	0.66	0.44					0.65		
**6 Presence of symptoms** α = 0.58; 6.5% Variance explained											
Whether patient was restless	2.8	1.3	0.30	0.51						0.83	
Whether patient had pain	3.0	1.3	0.46	0.40						0.76	
Whether patient had retained respiratory tract secretions	2.7	1.4	**0.65**	**0.28**	**0.42**					**0.47**	
**7 Support at actual time of death and afterwards** α = 0.59; 5.7% Variance explained											
Support at actual time of death	3.5	1.0		0.43							0.76
Sensitivity of HCT after death	3.9	0.8		0.43	**0.38**						**0.44**

Range of numeric item scorings: 0 to 4 (see Table 1). Although scaling differ between items, higher values are always associated with higher quality. Values in bold were critical and have been analysed individually in order to decide on item in−/exclusion; HCT = Health Care Team; ^a^ = items were deleted after analysis

### Inclusion of items

Items loading on two factors were allocated according to higher loadings (items “whether patient had retained respiratory tract secretions”, “whether discussion of giving fluids through a ‘drip’ took place”, and “sensitivity of health care team after death” or content-related conformity (“time of nurses to listen and discuss the patients’ condition”). The item on “whether ward was clean” was omitted from further analysis since there was no obvious content-related conformity to its factor; internal consistency of the *Spiritual and emotional support* subscale increased to 0.86 if it was deleted. The item on “whether patient died in the right place” was dropped from further analysis because its correlation with the *Environment* subscale was rather low; alpha increased to 0.81 if it was deleted. Although items on “whether patient had retained respiratory tract secretions” and “whether discussion of giving fluids through a ‘drip’ took place” had four critical values, they were not excluded because the expert panel rated their content consistent to their factors and the two to be indispensable components of QOC. Internal consistency for factors varied between α = 0.58 and α = 0.86 after the deletion of Items “whether ward was clean” and “whether patient died in the right place”. Table 4 shows the scale analysis based on the 26-item solution. Items “whether discussion of giving fluids through a ‘drip’ took place”, “whether discussion giving fluids through a ‘drip’ would have been helpful” and “emotional support to next-of-kin” showed only marginal critical values and therefore were kept for the final solution.

**Table 4:** Final scale analysis (after omission of Items “whether ward was clean” and “ Whether patient died in the right place”)

	Overall Cronbach’s Alpha = 0.86	
Subscales and short description of items	Cronbachs’Alpha if item deleted per scale	Item Scale Correlation
**1 Support and time of doctors and nurses** α = 0.85; 14.6% Variance explained		
Nursing care - medicines and comfortable position	0.82	0.68
Nursing care - personal care needs	0.82	0.68
Confidence and trust with the doctors	0.83	0.57
Time of doctors to listen and discuss the patient’s condition	0.82	0.69
Whether HCT did all they could to relieve pain	0.84	0.54
Whether HCT did all they could to control respiratory tract secretions	0.85	0.41
Confidence and trust in nurses	0.84	0.51
Whether HCT did all they could to relieve restlessness	0.83	0.56
Time of nurses to listen and discuss the patients’ condition	0.83	0.57
**2 Spiritual and emotional support** α = 0.86; 10.6% Variance explained		
Whether HCT met overall religious spiritual needs of patient	0.74	0.82
Whether HCT met overall religious spiritual needs of next-of-kin	0.75	0.80
Emotional support to next-of-kin	**0.87**	.62
**3 Information and decision-making** α = 0.61; 8.5% Variance explained		
Difficulty of explanations about the patient’s condition	0.50	0.54
Involvement in decision-making	0.43	0.55
Whether discussion giving fluids through a ‘drip’ would have been helpful	**0.62**	**0.28**
Whether discussion of giving fluids through a ‘drip’ took place	**0.62**	**0.34**
**4 Environment** α = 0.81; 8.2% Variance explained		
Whether the bed area had adequate privacy	0.81	0.68
Whether the bed area was comfortable	0.81	0.68
**5 Information about dying process** α = 0.68; 7.6% Variance explained		
Whether a discussion about what to expect during the dying process would have been helpful	0.51	0.55
Information about what to expect during the dying process of the patient	0.56	0.52
Information about the soon death of the patient	0.66	0.44
**6 Presence of symptoms** α = 0.58; 6.5% Variance explained		
Whether patient was restless	0.51	0.30
Whether patient had pain	0.46	0.40
Whether patient had retained respiratory tract secretions	**0.62**	**0.26**
**7 Support at actual time of death and afterwards** α = 0.59; 5.7% Variance explained		
Support at actual time of death		0.43
Sensitivity of HCT after death		0.43

Values in bold were critical and have been analysed individually in order to decide on item in−/exclusion; HCT = Health Care Team

### Reliability and validity

Overall internal consistency was good (α = 0.86). Mean scorings of items with a rating interval scale varied between 3.0 and 3.86, for dichotomous items between 1.8 and 3.9 (Table 3). ICC (1) for interrater reliability was 0.79 (CI95% = 0.6–0.9; F(37,38) = 8.5, *p* < .001). ICC (1, 2) for test-retest reliability was 0.85 (CI95% = 0.8–0.9; F(54,54) = 11.9, *p* < .001). Coefficients for convergent validity showed a medium correlation between POS and CODE-GER (*r* = − 0.41, *p* < .001).

### Correlation between Total-score, subscales and items of overall impressions

Mean Total-Score was 85.69 (SD = 14.17; range = 25–104). Correlations between items of overall impression and Total-Score were weak to moderate (r/rs = 0.36–0.67; *p* < .01). Concerning the subscales, factor 1 (support and time of doctors and nurses) showed the highest correlation (*r* = 0.72; *p* < .01) with items of overall impression, factor 6 (presence of symptoms) the lowest (rs = − 0.02) (Table 5).

**Table 5:** Correlations between overall impression items and the subscales as well as the Total-Score

			Subscales and Total-Score							
			1	2	3	4	5	6	7	Total-Score
Overall items; numbering corresponding to CODE-GER		N								
27a [1]	Whether patient was treated with respect and dignity by doctors	212	0.51^**^	0.39^**^	0.24^**^	0.14^*^	0.14^*^	0.09	0,53^**^	0.46^**^
27b [1]	Whether patient was treated with respect and dignity by nurses	223	0.49^**^	0.35^**^	0.14^*^	0.17^*^	0.22	0.16^*^	0.23^**^	0.36^**^
28 [2]	Whether next-of-kin was adequately supported	230	0.44^**^	0.38^**^	0.36^**^	0.14^*^	0.39^**^	−0.02	0.56^**^	0.44^**^
29 [1]	Whether next-of-kind would recommend ward to family/friends	227	0.72**	0.57**	0.32**	0.44**	0.29**	0.15*	0.63**	0.67**

1 = Pearson-Moment-Correlation; 2 = Spearman’s rank correlation coefficient; *p < 0.05; **p < 0.01; subscales: (1) support and time of doctors and nurses, (2) spiritual and emotional support, (3) information and decision-making, (4) environment, (5) information about dying process, (6) symptom presence, (7) support at actual time of death and afterwards

### Difficulty of questionnaire, strain caused by assessment and time for filling out

Difficulty of the questionnaire was rated rather low (M = 2.19; SD = 2.4; range = 0–10); and mean strain caused by the assessment was 4.05 (SD = 3.05; range = 0–10). Mean duration of the assessment was 43.15 min with a maximum of 240 min. Information for interrater and T2 is shown in Table 6.

**Table 6:** Time for filling out, difficulty and strain caused by assessment

	N	M	SD	Range
Time for filling out - Main Sample	229	43.2	34.8	10–240
Time for filling out - Interrater	38	34.9	22.6	10–120
Time for filling out - T2	55	27.8	18.7	8–90
Difficulty of the questionnaire - Main sample	235	2.2	2.4	0–10
Difficulty of the questionnaire - Interrater	38	2.0	2.0	0–8
Difficulty of the questionnaire - T2	54	2.3	2.4	0–10
Strain caused by assessment - Main sample	235	4.1	3.2	0–10
Strain caused by assessment - Interrater	38	3.6	3.4	0–10
Strain caused by assessment - T2	54	3.4	3.4	0–10

T2 = Participants of repeated measurement

## Discussion

We performed translation, cultural adaptation and psychometric validation of the CODE questionnaire for the German setting. Participants of this study were next-of-kin, mostly husband/wife/partner or children of the deceased patient, similar to previous CODE™ or ECHO-D studies [7, 27]. CODE-GER showed good psychometric properties. Content validity was achieved through the standardized translation process and cognitive interviews with next-of-kin, which led to minimal adaptions. Although overall internal consistency was relatively high, it varied between factors from satisfactory to good. However, as all factors cover meaningful contents, none of the factors were deleted from the final solution, as recommended by Schmitt [28]. Congruency of the Total-Score between two raters and over time was good; the same applies for convergent validity.

### Items of overall impression

Although correlations between items of overall impression and Total-Score were moderate, none of them had consistent correlations with all factors. Thus, the sole use of these items is not recommended.

### Comparison with previous data

The German and English versions of the CODE questionnaire are identical regarding the content and number of items referring to QOC, but they differ in the coding system of answering options, number of items used for scale formation and the number of subscales. While the English version includes 12 of its 28 core items distributed on 3 key composite scales (“Environment”, “Care” and “Communication”), the German version includes 26 of its 28 core items distributed on 7 subscales to form the Total-Score (Table 4). Consequently, the ranges of the Total-Score differ between the English and the German version. Furthermore, the only identical subscale between the two versions is the *Environment* subscale. Herein, internal consistencies of the subscales are comparable (CODE-GER α = 0.81); CODE™ α = 0.89) [7].

### Strengths of the study

High research quality was achieved by following strict translation and research guidelines. To date CODE™ data had been analysed on the basis of a priori assumptions on the relationship between items. A strength of this study was the use of PCA to reveal the underlying structure of the questionnaire without a priori assumptions and to reduce data, ensuring that the most important items were displayed.

The response rate of this study indicates feasibility of the opt-in model, although low, but similar to previous ECHO-D or CODE™ studies using opt-out models [7, 27, 29, 30]. The results for questionnaire difficulty and assessment strain were also comparable to a previous study [6]. The above results point out the feasibility of the questionnaire and support previous study results showing that next-of-kin are capable of evaluating the care of the dying patient.

### Limitations

That said, it should be remembered that information on characteristics of non-responders such as socioeconomic status was not available. Socioeconomic status might have both an impact on response as well as on difficulties and perceived strains. The non-responders may feel more burdened after their relative’s death than the participating study population. Thus, non-responders may have rated the questionnaire as more emotionally strainful than the study sample. Further limitations need to be considered when interpreting the results of the study. In addition, it is difficult to interpret test-retest reliability as it is not clear, whether the assessment of quality of care is a rather stable or unstable construct in an interval of 8 weeks. It is debatable whether the sample was representative of the hospital population. Most participants were German (89%) and, similar to previous findings, women with Christian affiliation [29]. As approximately 17% of patients in Germany have a migrant background and 5% of the German population are Muslim [31, 32], the low participation rate of these groups might indicate a cultural obstacle, either in caring of these patients and their next-of-kin or in our recruitment method.

Further research is necessary to determine whether specific items are more essential for the Total-Score than others. Moreover, cut-off values which indicate poor, moderate or high quality of care would add practical value.

As not all significant decisions for the final CODE-GER version were exclusively based on statistical values the factor solution needs to be examined in further studies. Therefore, we would recommend future studies to apply confirmatory factor analysis to quantify the goodness of fit of our factorial solution.

## Conclusion

This study is the first translation and validation of CODE™^.^ in another language. Correspondingly, it is the first examination of the construct of CODE™ in a different population. The results from the present study offer confirmation that CODE-GER is a reliable and valid instrument. Moreover, our study shows that by including 26 of 28 items into the psychometric analysis, 7 subscales emerge which considerably increase the informative value of the original CODE-Questionnaire. Our study therefore not only provides the first validated tool in German language to assess QOC of the dying in last few days. It also presents an advancement of the original questionnaire, increasing its potential for further surveys and research. Future studies are recommended applying confirmatory factor analysis to quantify the goodness of fit of our factorial solution. CODE-GER (additional file 1) is now ready to assess QOC of the dying and to identify areas for improvement, which then can lead to the development of purposive interventions.

## Supplementary information

**Additional file 1.** CODE-GER Questionnaire (in English language) with the five thematically arranged sections from the original CODE TM in order to facilitate the completion of the questionnaire for relatives.

## Data Availability

CODE-GER may be obtained from the authors of this study. The datasets used and/or analysed during the current study are available from A.R.V. or M.H. on reasonable request.

## Abbreviations

CODE: Care of the Dying Evaluation
CODE-GER: Care of the Dying Evaluation – German
EORTC: European Organisation for Research and Treatment of Cancer
POS: Palliative care Outcome Scale
ICC: Inter/Intra Class Correlation
M: Mean
SD: Standard Deviation
PC: Palliative care
QOC: Quality of Care
QoDD: Quality of Death and Dying
ECHO-D: Evaluation Care and Health Outcomes
MZ: Mainz
E: Erlangen
T1: First measurement point
T2: Second measurement point
PCA: Principal component analysis
r: Pearson’s correlation coefficient
rs: Spearman’s rank correlation coefficient
CI: Confidence interval
