# Supplementary material for: Assessment of the quality of end-of-life care: translation and validation of the German version of the “Care of the Dying Evaluation” (CODE-GER) - a questionnaire for bereaved relatives
Source: Health Qual Life Outcomes. 2020 Sep 22;18:311. doi: 10.1186/s12955-020-01473-2 (PMC7507719; doi:10.1186/s12955-020-01473-2)
Supplement: Supplementary file 1 — Additional file 1. CODE-GER Questionnaire (in English language) with the five thematically arranged sections from the original CODE TM in order to facilitate the completion of the questionnaire for relatives. [file 12955_2020_1473_MOESM1_ESM.pdf]

CODE-GER (English translation)

<u>Care Of the Dying Evaluation</u>

You have been sent this questionnaire as you are registered as the next of kin to a patient who died in (*insert organisation*). This is a questionnaire on the care received by people in the last hours and days of life and the experiences of their families and friends during that time. Although we know the patient's name, for convention, we have referred to them as 'patient' or 's/he' in the questionnaire.

We realise this questionnaire may bring back strong memories and emotions and that reading it for the first time may be difficult. You may wish to wait and find some place quiet to read the questionnaire. If you feel upset or distressed in any way, you do not have to continue with the questionnaire and can stop at any time.

Instructions for completion

We would like you to focus on the last **<u>two days</u>** of his/her life, when answering these questions.

As you go through the questionnaire, please follow the instructions and answer the questions by crossing the most appropriate box, like this:**[×]**

If you would rather not or cannot answer one of the questions, please go onto the next one.

Your answers will be treated strictly confidential. Individuals will not be identifiable in the reports we write.

Section A

A1 The care received from the nurses & doctors

These questions are concerned with the general care s/he received from the doctors and nurses and the environment in which this care was delivered. The questions apply to the last **two days** of his/her life and relates to the doctors and nurses (including healthcare assistants and / or care agency staff) who were most involved with his/her care during this time.

Please look at the following statements and cross [×] the answer box that corresponds most with your opinion.

1. There was enough help available to meet his/her personal care needs, such as washing, personal hygiene and toileting needs.

- ☐ Strongly agree
- ☐ Agree
- ☐ Neither agree nor disagree
- ☐ Disagree
- ☐ Strongly disagree

2. There was enough help with nursing care, such as giving medicines and helping him/her find a comfortable position in bed.

- ☐ Strongly agree
- ☐ Agree
- ☐ Neither agree nor disagree
- ☐ Disagree
- ☐ Strongly disagree

3. The bed area and surrounding environment was comfortable for him/her.

- ☐ Strongly agree
- ☐ Agree
- ☐ Neither agree nor disagree
- ☐ Disagree
- ☐ Strongly disagree

4. The bed area and surrounding environment had adequate privacy for him/her.

- ☐ Strongly agree
- ☐ Agree
- ☐ Neither agree nor disagree
- ☐ Disagree
- ☐ Strongly disagree

5. Did you have confidence and trust in the nurses who were caring for him/her?

- ☐ Yes, in all of them
- ☐ Yes, in some of them
- ☐ No, not in any of the nurses

6. Did you have confidence and trust in the doctors who were caring for him/her?

- ☐ Yes, in all of them
- ☐ Yes, in some of them
- ☐ No, not in any of the doctors

7. The nurses had time to listen and discuss his/her condition with me.

- ☐ Strongly agree
- ☐ Agree
- ☐ Neither agree nor disagree
- ☐ Disagree
- ☐ Strongly disagree

8. The doctors had time to listen and discuss his/her condition with me.

- ☐ Strongly agree
- ☐ Agree
- ☐ Neither agree nor disagree
- ☐ Disagree
- ☐ Strongly disagree

Please feel free to comment, if you wish to, on any aspect of <u>questions 1-8</u>:

A2 The control of pain & other symptoms

These questions are concerned with the symptoms s/he had and the care s/he received during the last two days of his/her life. Please answer tick the answer that corresponds most with your opinion.

9. In your opinion, during the last two days, did s/he appear to be in pain?

- ☐ Yes, all of the time
- ☐ Yes, some of the time
- ☐ No

10. In your view, did the doctors and nurses do enough to help relieve the pain?

- ☐ Yes, all of the time
- ☐ Yes, some of the time
- ☐ No, not at all
- ☐ Not applicable, s/he was not in pain

11. In your opinion, during the last two days, did s/he appear to be restless?

- ☐ Yes, all of the time
- ☐ Yes, some of the time
- ☐ No

12. In your view, did the doctors and nurses do enough to help relieve the restlessness?

- ☐ Yes, all of the time
- ☐ Yes, some of the time
- ☐ No, not at all
- ☐ Not applicable, s/he was not restless

13. In your opinion, during the last two days, did s/he appear to have a 'noisy rattle' to his/her breathing?

- ☐ Yes, all of the time
- ☐ Yes, some of the time
- ☐ No

14. In your view, did the doctors and nurses do enough to help relieve the 'noisy rattle' to his/her breathing?

- ☐ Yes, all of the time
- ☐ Yes, some of the time
- ☐ No, not at all
- ☐ Not applicable, s/he was not restless

Please feel free to comment, if you wish to, on any aspect of questions 9-14:

A3 Communication with the healthcare team

The following questions are about the communication that you, your family members and friends received from the healthcare team who were most involved with his/her care in the last **two days** of his/her life. By **'healthcare team'**, we mean the doctors, the nurses and any other member of staff who may have been involved in his/her care such as a social worker or a chaplain

15. During the last two days, how involved were you with the decisions about his/her care and treatment?

- ☐ Very involved
- ☐ Fairly involved
- ☐ Not involved

16. Did any of the healthcare team discuss with you whether giving fluids through a 'drip' would be appropriate in the last two days of life?

- ☐ Yes
- ☐ No
- ☐ Don´t know

17. Would a discussion about the appropriateness of giving fluids through a 'drip' in the last two days of life have been helpful?

- ☐ Yes
- ☐ No
- ☐ Not applicable, we had these types of discussions

18. Did the healthcare team explain his/her condition and/or treatment in a way you found easy or difficult to understand?

- ☐ Very easy
- ☐ Fairly easy
- ☐ Fairly difficult
- ☐ Very difficult
- ☐ They did not explain his/her condition or treatment to me

Please feel free to comment, if you wish to, on any aspect of questions 15-18:

A4 The emotional & spiritual support provided by the healthcare team

The following questions are about the emotional and spiritual support that was provided to you and your family member or friend by the healthcare team in the last **two days** of his/her life. By '**spiritual support**', we mean support relating to important personal beliefs. These beliefs may be connected with a specific religion but may also be personal beliefs about what life means, what provided you or your family member / friend with hope and helped you cope.

19. How would you assess the overall level of emotional support given to you by the healthcare team?

- ☐ Excellent
- ☐ Good
- ☐ Fair
- ☐ Poor

20. Overall, his/her religious or spiritual needs were met by the healthcare team.

- ☐ Strongly agree
- ☐ Agree
- ☐ Neither agree nor disagree
- ☐ Disagree
- ☐ Strongly disagree

21. Overall, my religious or spiritual needs were met by the healthcare team.

- ☐ Strongly agree
- ☐ Agree
- ☐ Neither agree nor disagree
- ☐ Disagree
- ☐ Strongly disagree

Please feel free to comment, if you wish to, on any aspect of questions 19-21:

A5. The circumstances surrounding his/her death

The following questions are about the circumstances surrounding his/her death, and your feelings about the way in which the healthcare team treated you and your family member at this time. By **'healthcare team'**, we mean the doctors, the nurses and any other member of staff who may have been involved in his/her care such as a social worker or a chaplain

22. Before s/he died, were you told s/he was likely to die soon?

- ☐ Yes
- ☐ No

<u>If 'Yes'</u>, who told you s/he was likely to die soon?

- ☐ Doctor
- ☐ Nursing team
- ☐ Other member of staff: ______________________

23. Did a member of the healthcare team talk to you about what to expect when s/he was dying (e.g. symptoms that may arise)?

- ☐ Yes
- ☐ No

24. Would a discussion about what to expect when s/he was dying have been helpful?

- ☐ Yes
- ☐ No
- ☐ Not applicable, we had these types of discussions

25. I was given enough help and support by the healthcare team at the actual time of his/her death.

- ☐ Strongly agree
- ☐ Agree
- ☐ Neither agree nor disagree
- ☐ Disagree
- ☐ Strongly disagree

26. After s/he had died, did individuals from the healthcare team deal with you in a sensitive manner?

- ☐ Yes
- ☐ No
- ☐ Not applicable, I didn´t have any contact with the healthcare team

Please feel free to comment, if you wish to, on any aspect of <u>questions 22-26:</u>

____

____

____

____

____

____

____

____

____

____

____

____

____

____

____

____

____

____

Section B
Overall impressions

The following questions are about your overall impression of the care s/he received in the last **two days** of life and your experiences during that time.

27. How much of the time was s/he treated with respect and dignity in the last two days of life?

Please answer for both doctors and nurses

	Doctors	Nurses
Always	☐	☐
Most of the time	☐	☐
Some of the time	☐	☐
Never	☐	☐
Don´t know	☐	☐

28. Overall, in your opinion, were you adequately supported during his/her last two days of life?

☐ Yes
☐ No

29. How likely are you to recommend the ward to friends and family?

Extremely likely ☐
Likely ☐
Neither likely nor unlikely ☐
Unlikely ☐
Extremely unlikely ☐
Don’t know ☐

Please feel free to comment, if you wish to, on any aspect of questions 27-29:

Section C:
Optional Items

O 1. In your opinion, how clean was the ward area that s/he was in?

Very clean ☐
Fairly clean ☐
Not at all clean ☐

O 2. In your opinion did s/he die in the right place?

Yes, it was the right place ☐
No, it was not the right place ☐
Not sure ☐
Don’t know ☐

Although the focus of this questionnaire has very much been on his/her last days of life, we appreciate there may be other aspects of care or support prior to this time, which you wish to feedback. Please feel free to comment, if you wish to, on any aspect of the overall care and support received:

Section D

Information about you and the patient

We would like to know a little more about you and the patient. This will help us make further use of the information you give us and will remain strictly confidential.

30. What was your relationship to him/her?

Were you his/her:

- ☐ Husband/Wife/Partner
- ☐ Daughter/Son
- ☐ Sister/Brother
- ☐ Daughter-in-law/ Son-in-law
- ☐ Mother/Father
- ☐ Friend
- ☐ Neighbour
- ☐ Other (please specify):

____

31. What is your age?

- ☐ 18-19
- ☐ 20-29
- ☐ 30-39
- ☐ 40-49
- ☐ 50-59
- ☐ 60-69
- ☐ 70-79
- ☐ 80+

32. What is your nationality?

____

33. Migration background?

- ☐ Yes, Country:______________________
- ☐ No

34. Are you:

- ☐ Female
- ☐ Male

____

35. What is your religious affiliation?

- ☐ Protestant
- ☐ Roman Catholic
- ☐ Muslim
- ☐ No affiliation
- ☐ Other (please specify):

____

36. Please cross all the illnesses s/he had in the last days of life.

- ☐ Cancer
- ☐ Heart failure
- ☐ COPD
- ☐ Kidney disease
- ☐ Dementia
- ☐ Motor Neurone Disease
- ☐ Stroke
- ☐ Don´t know
- ☐ Others (please specify):

____

37. What was his / her age when s/he died?

- ☐ 18-19
- ☐ 20-29
- ☐ 30-39
- ☐ 40-49
- ☐ 50-59
- ☐ 60-69
- ☐ 70-79
- ☐ 80+

38. Which nationality did s/he have?

____

39. Migration background?

- ☐ Yes, Country:______________________
- ☐ No

40. What was his/her religious affiliation?

- ☐ Protestant
- ☐ Roman Catholic
- ☐ Muslim
- ☐ No affiliation
- ☐ Other (please specify):

____

41. On which ward did s/he die?

- ☐ Curative ward
- ☐ Intensive care unit
- ☐ Palliative care unit
- ☐ Other (please specify)

____

- ☐ Don´t know

42. How many days had he/she been on the ward where she died?

____

Thank you very much for taking the time to complete this questionnaire.

We would be very grateful if you could return the questionnaire to us in the stamped address envelope provided, or post to:
